# Novel Likely Pathogenic Variants Identified by Panel-Based Exome Sequencing in Congenital Cataract Patients

**DOI:** 10.1155/2021/3847409

**Published:** 2021-11-17

**Authors:** Doudou Chen, Tao Yang, Siquan Zhu

**Affiliations:** ^1^Eye School of Chengdu University of Traditional Chinese Medicine, Chengdu 610075, Sichuan, China; ^2^Department of Ophthalmology, Ineye Hospital of Chengdu University of Traditional Chinese Medicine, Chengdu 610032, Sichuan, China; ^3^Key Laboratory of Sichuan Province Ophthalmopathy Prevention and Cure and Visual Function Protection, Chengdu University of Traditional Chinese Medicine, Chengdu 610032, Sichuan, China; ^4^Department of Traditional Chinese Medicine, Beijing Tian Tan Hospital, Capital Medical University, Beijing 100050, China; ^5^Department of Ophthalmology, Beijing Anzhen Hospital, Capital Medical University, Beijing 100006, China

## Abstract

**Purpose:**

To identify likely pathogenic variants in three families with congenital cataracts via panel-based exome sequencing.

**Methods:**

A panel containing 153 genes associated with congenital cataracts was designed. Genes were selected through reference to databases including the Human Gene Mutation Database (HGMD), Online Mendelian Inheritance in Man (OMIM), Genetic Home Reference, and the latest peer-reviewed publications on the genetics of hereditary cataracts. Panel-based exome sequencing was performed with the Illumina HiSeq X-Ten platform, and then the identified variants were confirmed with Sanger sequencing and evaluated according to the American College of Medical Genetics and Genomics (ACMG) criteria.

**Results:**

Three likely pathogenic variants were found. A novel *CRYBB2*: c.230G > T p.G77V variant was identified in family A, a novel *CRYBB2*: c.230G > A p.G77D variant was identified in family B, and a novel *CRYGD*: c.475delG p.A159Pfs∗9 variant was identified in family C.

**Conclusion:**

Panel-based exome sequencing revealed three likely pathogenic variants in three unrelated Chinese families with congenital cataracts. These data expand the genetic spectrum associated with congenital cataracts.

## 1. Introduction

Autosomal dominant congenital cataracts (ADCCs) are congenital eye abnormalities with phenotypic variability from congenital cataracts, and some but not all lead to early visual impairment. ADCCs may impede visual development by affecting lens transparency and may develop before or after birth. Hereditary factors can account for 10–25% of all congenital cataracts [1]. Congenital cataracts are responsible for 5–20% of blindness in individuals living in developed countries and 22–30% in those living in developing countries [2].

Congenital or developmental cataracts demonstrate significant genotypic and phenotypic variability. Recent studies have identified an increasing number of loci and genes associated with congenital cataracts. Until May 12, 2021, the Online Mendelian Inheritance in Man (OMIM) database included 42 non-syndrome-related genes and 27 syndrome-related genes, including genes encoding *α*-, *β*-, and *γ*-crystallins, such as *CRYAA* (NG_009823.1), *CRYBB2* (NG_009827.1), and *CRYGD* (NG_008039.1); *α*-connexins, such as *GJA3* (NG_016399.1) and *GJA8* (NG_016242.1); other lens membrane or cytoskeletal proteins genes, such as *MIP* (NG_021397.2) and *BFSP2* (NG_ and 012425.1); several transcription factors, such as *HSF4* (NG_009294.1) and *PITX3* (NG_008147.1); and an expanding group of functionally divergent genes, including *EPHA2* (NG_021396.1), *TDRD7* (NG_028984.1), and *FYCO1* (NG_031955.1) [1, 3–5].

Previously, Sanger sequencing combined with linkage analysis was used to identify the pathogenic genes in hereditary cataracts. Currently, panel-based targeted exome sequencing is a practical method for identifying monogenic diseases. A specific Hereditary Eye Disease Enrichment Panel developed by MyGenostics (Beijing, China) has been used to identify a variety of genetic eye diseases.

## 2. Materials and Methods

### 2.1. Participants

Three Han Chinese families were recruited. One hundred unrelated healthy subjects were also recruited from the physical examination centre. A detailed medical history of the participants was obtained. All participants underwent slit-lamp ophthalmoscopy, biometry, and best-corrected vision acuity (BCVA) as measured with ETDRS linear optotypes/picture symbols. All members of the control group had no anterior segment disease, no fundus disease, no refractive error, no history of trauma, and no family history of genetic disease. The DNA samples were extracted using a DNA Blood Midi Kit (Qiagen, Hilden, Germany). Ethics committee approval was obtained from the Institutional Review Board of Chengdu University of Traditional Chinese (CMEC2010-22), and written informed consent was provided by all participants.

### 2.2. Targeted Gene Enrichment and Sequencing

One hundred fifty-three genes (Table S1) associated with congenital cataracts were selected via a gene capture strategy using a GenCap custom enrichment kit (MyGenostics, China) following the manufacturer's instructions [6]. The capture probe was designed to cover all exon regions of the target gene without repeating regions. The extracted DNA was eluted and amplified, polymerase chain reaction (PCR) products were purified with SPRI beads (Beckman, USA), and the enriched library was read and sequenced with the Illumina HiSeq X-Ten system.

### 2.3. Bioinformatics Analysis and Variant Selection

Panel-based targeted exon sequencing (TES) was performed on the probands and their parents. The raw data were saved in FASTQ format. Illumina sequencing adapters and low-quality reads (<80 bp) were filtered by Cutadapt [7]. After quality control, the clean reads were mapped to the UCSC hg38 human reference genome using Burrows–Wheeler Aligner (BWA) [8]. Duplicated reads were removed using Picard tools, and only uniquely mapped reads were used for variation detection. Single-nucleotide polymorphisms (SNPs) and indels were detected by GATK [9]. The variants were further subjected to annotation via ANNOVAR [10], searches in multiple databases, such as 1000 Genomes, ESP6500.exon.program, dbSNP, gnomAD, InterVar, SPIDEX, REVEL, MCAP, Inhouse (MyGenostics), and Human Gene Mutation Database (HGMD), and prediction analysis via SIFT [11], PolyPhen-2 [12], MutationTaster [13], and GERP++ [14, 15]. The interpretation and evidence grading of the variants were performed according to the latest edition of the standards and guidelines for the interpretation of gene variation issued by the American College for Medical Genetics and Genomics (ACMG) [4, 16–18].

### 2.4. Validation of Candidate Variants and Segregation

Regions harbouring point variants were amplified. Polymerase chain reaction (PCR) and Sanger sequencing were used to verify putative variants in all participants and controls. Purified PCR products were sequenced using an ABI 3730 Automated Sequencer (PE Biosystems, USA). PCR primers were designed by Invitrogen, Shanghai, China, as follows: *CRYBB2* forward primer (5′-GGCCCCCTCACCCATACTCA-3′) and reverse primer (5′-CTTCCCTCCTGCCTCAACCTAATC-3′); *CRYGD* forward primer (5′-GCTTTTCTTCTCTTTTTATTTCTGG-3′), reverse primer (5′-AAGAAAGACACAAGCAAATCAGT-3′).

### 2.5. Bioinformatics Analysis

Pymol shows the protein structure of wild type and variant. Additionally, the potential functional influence from the variant was calculated by PolyPhen-2 and SIFT. Kyte–Doolittle algorithm of ProtScale was adopted to analyze the hydrophobic properties and chemical parameters of protein.

## 3. Results

The clinical results of the three families are shown in Table 1.

### 3.1. Family A

**Figure 1:**
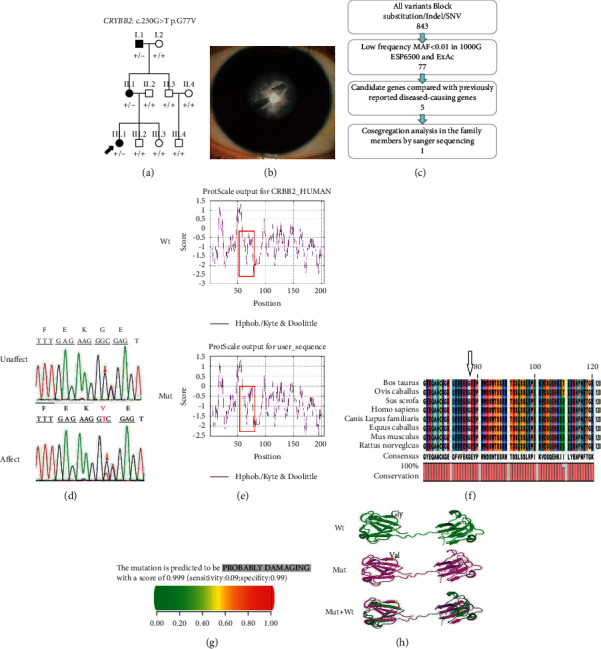
(a) Pedigree of ADCC family A Squares and circles indicate males and females, respectively. The solid black arrow indicates the proband (III.1). (b) A photograph of the proband's (III.1) lens is shown. (c) Schematic representation of the filter strategies employed in our study. (d) Sanger sequencing results. (e) Hydropathic characteristics caused by changes in the variant protein. (f) Multiple protein sequence alignment. (g) The score obtained from PolyPhen-2 analysis was 0.999. (h) The structures of the homomeric WT and variant *CRYBB2* c.230G > T; p.G77 V proteins were modelled in PyMOL.

The pedigree chart of family A is shown in Figure 1(a). The proband (III.1) presented with bilateral complete cataracts at birth (Figure 1(b)). She underwent binocular cataract surgery sequentially without implantation of an intraocular lens at 2 months. Postoperatively, she was corrected with glasses. She underwent bilateral intraocular lens implants at the age of 2 years. Her BCVA at the age of 4 years was 1.0 logMAR (Snellen equivalent 20/200) in the right eye and 1.0 logMAR (Snellen equivalent 20/200) in the left eye as measured with ETDRS picture symbols. Her anterior segment had no other abnormalities except for cataracts and partial iris loss. This partial iris loss may have been caused by postoperative complications. The fundus cup-to-disc ratio was 0.2 in the right eye and 0.2 in the left eye, and no abnormalities were found in the fundus periphery. She did not have an afferent pupillary defect. Her motility was full, with esotropia. Nystagmus secondary to low vision was present. Her mother (II.1) also presented with bilateral complete cataracts at birth. She underwent bilateral cataract surgery sequentially with intraocular lens implantation at the age of 2 years. There was no refractive correction after the operation. Her BCVA at the age of 32 years was 1.3 logMAR (Snellen equivalent 20/400) in the right eye and 1.0 logMAR (Snellen equivalent 20/200) in the left eye as measured with ETDRS linear optotypes. Except for cataracts, no other abnormalities were found in her anterior segment. She did not have an afferent pupillary defect, but she had esotropia and nystagmus that may have been triggered by low vision. On fundus examination, the binocular cup-to-disc ratio was 0.3, and no abnormalities were found in the fundus periphery. Her grandfather (I.1) also presented with complete bilateral congenital cataracts at birth. He underwent cataract surgery sequentially without intraocular lens implantation at the age of 2 years. There was no refractive correction after the surgery. His BCVA at the age of 55 years was 1.7 logMAR (Snellen equivalent 20/1000) in the right eye and 1.6 logMAR (Snellen equivalent 20/800) in the left eye as measured with ETDRS linear optotypes. Except for irregular pupils that may have been caused by surgical complications, there were no other abnormalities. He did have an afferent pupillary defect that may have been associated with partial atrophy of the optic nerve. He also had esotropia and nystagmus secondary to low vision. On fundus examination, the binocular cup-to-disc ratio was 0.4, and the optic disc was pale. Optical coherent fundus examination revealed that the optic disc nerve fibre layer was thin with some optic nerve atrophy. However, the intraocular pressure was within the normal range.

### 3.2. Family B

**Figure 2:**
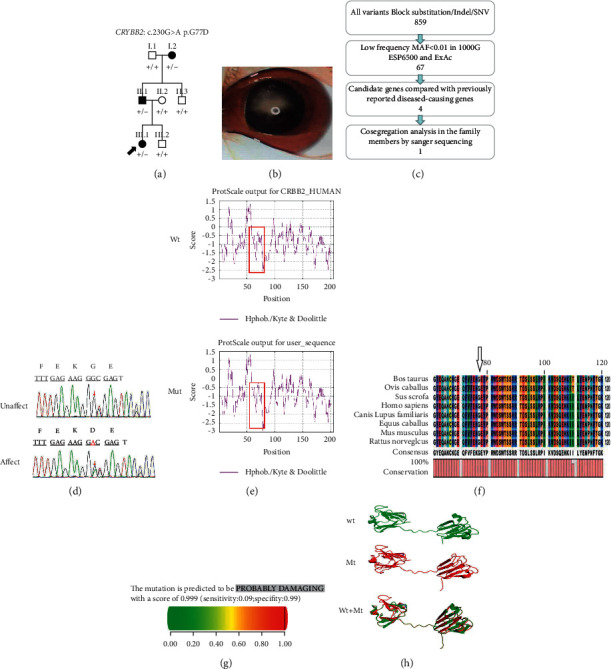
(a) Pedigree of ADCC family B The solid black arrow indicates the proband (III.1). (b) A photograph of the proband's (III.1) lens is shown. (c) Schematic representation of the filter strategies employed in our study. (d) Sanger sequencing results. (e) hydropathic characteristics caused by changes in the variant protein. (f) Multiple protein sequence alignment. (g) The score obtained from PolyPhen-2 analysis was 0.999. (h) The structures of the homomeric WT and variant *CRYBB2*: c.230G > A; p.G77D proteins were modelled in PyMOL.

The pedigree chart of family B is shown in Figure 2(a). The proband (III.1) presented with bilateral nuclear cataracts at the age of 2 months (Figure 2(b)), and she underwent bilateral cataract surgery sequentially with intraocular lens implantation at the age of 2 years. After the operation, she was corrected with glasses. Her BCVA at the age of 2 years was 1.0 logMAR (Snellen equivalent 20/200) in the right eye and 0.7 logMAR (Snellen equivalent 20/100) in the left eye as measured with ETDRS picture symbols. Her anterior segment was normal except for cataracts. She did not have an afferent pupillary defect, and the cup-to-disk ratio of both eyes was 0.3 on fundus examination. Her motility was full, without strabismus and nystagmus. Her father (II.1) was found to squint frequently at the age of 2 years. He was diagnosed with binocular nuclear cataracts and underwent sequential bilateral cataract surgery combined with intraocular lens implantation. His BCVA at the age of 30 years was 0.4 logMAR (Snellen equivalent 20/50) in the right eye and 0.7 logMAR (Snellen equivalent 20/100) in the left eye as measured with ETDRS linear optotypes. His anterior segment had no other abnormalities except for cataracts. Fundus examination of the binocular cup-to-disk ratio was 0.2, and there were no other abnormalities. He did not have an afferent pupillary defect. His motility was full. The grandmother did not have any abnormalities at birth, but her family discovered that she had poor vision in both eyes at the age of 4 years. The grandmother (I.2) also presented with binocular nuclear cataracts. She underwent sequential bilateral cataract surgery with intraocular lens implantation at the age of 4 years. Her BCVA at the age of 55 years was 0.7 logMAR (Snellen equivalent 20/100) in the right eye and 1.0 logMAR (Snellen equivalent 20/200) in the left eye as measured with ETDRS linear optotypes. Her anterior segment had no other abnormalities except for cataracts. Fundus examination revealed a cup-to-disk ratio of 0.2 for both eyes and no other abnormalities. She did not have an afferent pupillary defect. Her motility was full, without strabismus and nystagmus.

### 3.3. Family C

**Figure 3:**
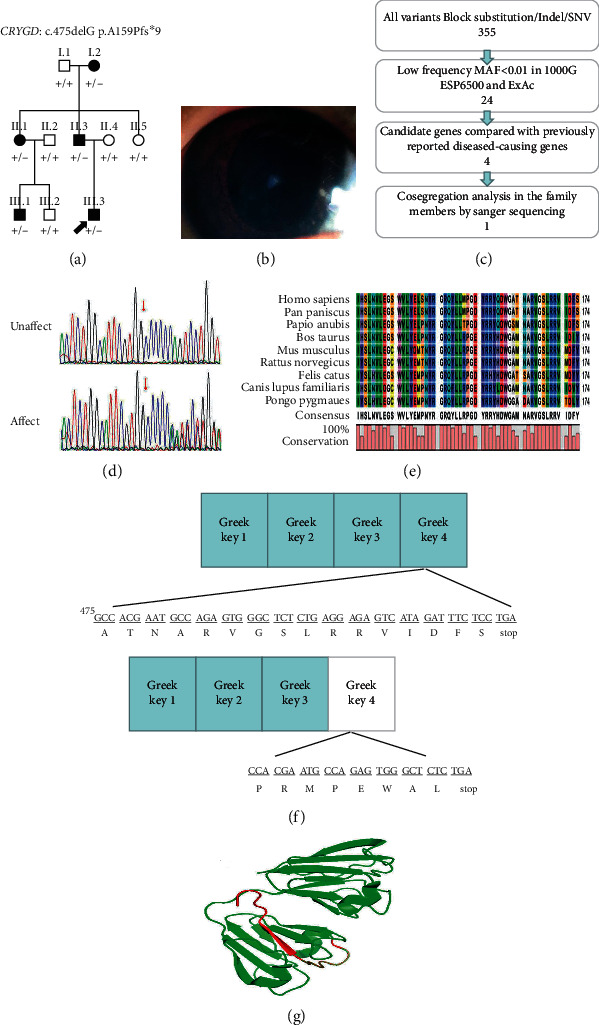
(a) Pedigree of ADCC family C The solid black arrow indicates the proband (III.3). (b) Photographs of the proband's (III.3) lens are shown. (c) Schematic representation of the filter strategies employed in our study. (d) Sanger sequencing results. (e) Multiple protein sequence alignment. (f) The identified variant, *CRYGD*: c.475delG; p.A159Pfs∗9, generated a frameshift and premature termination from a position 9 codons downstream (p.A159Pfs∗9) of the variant; these alterations resulted in a protein that was reduced to half the length of the full-length protein. (g) The structures of the *CRYGD* proteins were modelled in PyMOL.

The pedigree chart of family C is shown in Figure 3(a). The proband (III.3) gradually presented with binocular nuclear cataracts within one year after birth (Figure 3(b)). He underwent sequential binocular cataract surgery at the age of 3 years. After the operation, he did not undergo any vision correction procedure. His BCVA at the age of 3 years was 1.0 logMAR (Snellen equivalent 20/200) in the right eye and 1.0 logMAR (Snellen equivalent 20/200) in the left eye as measured with ETDRS picture symbols. His anterior segment had no abnormalities except for cataracts. Fundus examination revealed that the cup-to-disk ratio of both eyes was 0.3, and he did not have an afferent pupillary defect. His motility was full, with esotropia. Nystagmus secondary to low vision was noted. His father (II.2) gradually developed binocular nuclear cataracts within five years after birth, and he underwent sequential binocular cataract surgery at the age of 5 years. After the operation, he did not undergo any vision correction procedure. His BCVA at the age of 30 years was 0.5 logMAR (Snellen equivalent 20/63) in the right eye and 0.4 logMAR (Snellen equivalent 20/50) in the left eye as measured with ETDRS linear optotypes. His anterior segment had no other abnormalities except for cataracts. Fundus examination revealed that the cup-to-disk ratio of both eyes was 0.2. His mobility was full, and he did not have an afferent pupillary defect. He also had mild esotropia and nystagmus that may have been secondary to low vision. The grandmother (I.2) also presented with bilateral nuclear cataracts at the age of 2 years and underwent cataract extraction without intraocular lens implantation. Her BCVA at the age of 65 years was 1.7 logMAR (Snellen equivalent 20/900) in the right eye and 1.6 logMAR (Snellen equivalent 20/800) in the left eye as measured with ETDRS linear optotypes. Her anterior segment had no other abnormalities except for cataracts. Fundus examination revealed that the cup-to-disk ratio of both eyes was 0.2. Additionally, she had esotropia with nystagmus that may have been secondary to low vision. Another family member (II.1) was also diagnosed with congenital cataracts and underwent cataract surgery at the age of 4 years. Her BCVA at the age of 33 years was 0.7 logMAR (Snellen equivalent 20/100) in the right eye and 1.0 logMAR (Snellen equivalent 20/200) in the left eye as measured with ETDRS linear optotypes. Fundus examination revealed that the binocular cup-to-disk ratio was 0.2, without strabismus or nystagmus, and pupil defects. Another family member (III.1) was also diagnosed with congenital cataracts and underwent cataract surgery at the age of 4 years. His BCVA at the age of 11 years was 1.0 logMAR (Snellen equivalent 20/200) in the right eye and 1.0 logMAR (Snellen equivalent 20/200) in the left eye as measured with ETDRS linear optotypes. However, except for cataracts, he had no other abnormalities in the anterior segment, no strabismus or nystagmus, no pupil defects, and had a cup-to-disk ratio of 0.2 (fundus examination).

### 3.4. Sequencing Results and Biological Analysis

Sequencing results and biological analysis are described in the supplementary materials (available here), and sequencing data statistics are shown in Table 2. The predictive analysis of variant function is presented in Table 3, and candidate variants are shown in Table S2. The sequencing chromatograms of the three family members are shown in supplementary outcomes 1(Sanger A, Sanger B, and Sanger C) and the sequencing biological analysis results of the three families are shown in supplementary outcomes 2.

## 4. Discussion

In this study, we used panel-based targeted exome sequencing to identify three novel likely pathogenic variants in three unrelated Chinese families with congenital cataracts. The panel was designed based on a consensus of experts on the genetic diagnosis of eye diseases in July 2018 and by referring to the OMIM database, HGMD, and clinical data. To date, 356 genes associated with syndromic and nonsyndromic cataracts and nearly 50 disease-causing genes that are related to isolated cataracts have been identified (http://cat-map.wustl.edu/). The pathogenicity of these variant sites was analysed strictly following the ACMG guidelines [17].

*CRYBB2*:c.230G > T(p.G77V) caused family A to acquire complete cataracts. *CRYBB2*:c.230G > A(p.G77D) caused family B to acquire irregular nuclear cataracts. The p.G77V variant slightly increases hydrophobicity and has a mild effect on the protein structure. In addition, the variant alters a tyrosine corner, which is critical for the stability of the N-terminal domain and may lead to changes in the intermolecular action, solubility and stability of CRYBB2. The p.G77D variant causes the hydrophobicity of the variant protein to be lower than that of the wild-type protein and immense changes in charge and size that may also affect the protein's correct folding and stability. There are many intermolecular contacts between domains in CRYBB2. Both of these variants may disrupt the dimerization of the CRYBB2 protein or inhibit its binding to other lens-soluble proteins. These changes may destroy the microstructure of the lens, increase light scattering, and eventually cause the lens to become opaque. *CRYBB2* contains six exons, with the start of translation beginning in the second exon. Exons 3 to 6 each encode one Greek key motif (GKM). The variants in these two families are located in exon 4, which may cause the destruction of the corresponding GKM. At present, a total of 47 *CRYBB2* variants have been reported, 27 of which are unique. The p.W151C variant disrupts the solubility of *β*B2-crystallin and causes abnormal aggregation of the protein to form membrane cataracts [19]. The p.Q155X variant causes a partially unfolded structure and decreased structural order, inhibiting interactions with other proteins [20]. The p.A188H variant impairs the dimerization of the CRYBB2 protein by establishing a new hydrogen bond, thereby leading to lens opacity [21]. The p.D128V variant changes the hydrophobicity and charge of the random coil region between amino acids 126–139 of the CRYBB2 protein [22]. The p.W151C variant identified in an Indian family was predicted to disrupt the fourth GKM and increase the protein's hydrophobicity, thereby leading to the formation of cataracts [23]. Congenital cataracts have complex genetic and clinical heterogeneity [19]. The variants in these two families may be a susceptible mutation site, but further verification is needed.

Patients in family C developed bilateral nuclear cataracts within 1 to 5 years after birth. The identified variant, c.475delG located in the last exon of *CRYGD*, generated by a frameshift variant located 9 codons downstream that resulted in premature termination (p.A159Pfs∗9), resulted in a protein that was reduced to half the length of the full-length protein. In eukaryotes, mRNAs that contain premature stop codons can be detected and destroyed by an mRNA monitoring mechanism called nonsense-mediated decay (NMD). However, it usually occurs only when the nonsense codon is more than 50 nucleotides upstream of the last exon junction [24, 25]. Due to the variant p.A159Pfs∗9 is located at the end of the last exon in *CRYGD*, it should escape from NMD, resulting in translation of the truncated protein. In addition, the *CRYGD* gene contains four GKMs, each of which is approximately forty amino acids long. Two similar GFM motifs form a structural domain, resulting in a total of two structural domains. The specificity of domain interface interactions is likely important for preventing incorrect associations in the lens' nucleus due to high protein concentrations. We speculate that the p.A159Pfs∗9 in family C removes the last beta strand of the fourth GKM, destabilizing the fourth GKM severely and hence the entire domain. This protein probably does not fold normally, even during synthesis. This would destabilize the protein severely by eliminating a required part of the fourth GKM, which would bind to alpha-crystallin until it is overwhelmed and then either become toxic to the lens cell or form aggregates large enough to scatter light. A polypeptide or protein with a complete primary structure can function only if folded correctly to form the correct spatial structure. Changes in protein properties caused by variants may affect their functions and interactions with other proteins, leading to cataracts. Approximately 59 *CRYGD* variants have been documented to be associated with congenital cataracts, 25 of which are unique, and the remainder are recurrent. Most of these reported variants are single-base variants. p.G165fs is the only frameshift variant that has been previously reported [26]. In this research, the *CRYGD*: p.A159Pfs∗9 we discovered in this family C is a novel frameshift variant. However, there is no report on the monomerization and metabolic activities of gamma-crystallins. There is also a need to explore animal models to elucidate the underlying molecular mechanism by which CRYBB2 and CRYGD variants contribute to cataracts.

## 5. Conclusions

In this study, we identified three novel likely pathogenic variants, *CRYBB2*: c.230G > T (p.G77V), *CRYBB2*: c.230G > A (p.G77D), and *CRYGD*: c.475delG (p.A159Pfs∗9), in three unrelated Chinese families with hereditary cataracts. These data expand the genetic spectrum associated with congenital cataracts. The use of panel sequencing may provide a basis for the genetic diagnosis of congenital cataracts in patients with a family history and meet patients' needs through genetic counselling.

## Figures and Tables

**Table 1:** Clinical information of patients from three families.

	Patient	BCVA (R/L) LogMAR	Type of cataracts	Age of removal of cataract	IOL vs. aphakia	Fundus anomalies	Refractive error at age examined (R/L)	Nystagmus	Strabismus subtype
Family A	III.1	1.0/1.0	Bilateral complete cataract	2 months	IOL	No	+6.00DS/-2.00DC×10, +5.50DS/-1.00DC×140	Ophthalmic nystagmus	Esotropia
	II.1	1.3/1.0	Bilateral complete cataract	2 years	IOL	No	+1.00DS/-0.50DC×60, −2.250DS/-1.00DC×80	Ophthalmic nystagmus	Esotropia
	I.1	1.7/1.6	Bilateral complete cataract	2 years	aphakia	Optic atrophy	+19.00DS/-0.50DC×60, +22.50DS/-1.50DC×30	Ophthalmic nystagmus	Esotropia

Family B	III.1	1.0/0.7	Bilateral nuclear cataract	2 months	IOL	No	NA	No	No
	II.1	0.4/0.7	Bilateral nuclear cataract	2 years	IOL	No	+2.00DS/-1.50DC×170, +1.50DS/-1.50DC×110	No	No
	I.1	0.7/1.0	Bilateral nuclear cataract	4 years	IOL	No	+5.00DS/-1.75DC×50，+5.50DS/-1.00DC×110	No	No

Family C	III.3	1.0/1.0	Bilateral nuclear cataract	3 years	IOL	No	+4.50DS/-1.00DC×5，+6.00DS/-2.00DC×160	Ophthalmic nystagmus	Esotropia
	II.2	0.5/0.4	Bilateral nuclear cataract	5 years	IOL	No	+1.25DS/-0.75DC×130, +2.00DS/-1.50DC×90	Ophthalmic nystagmus	Esotropia
	I.2	1.7/1.6	Bilateral nuclear cataract	6 years	aphakia	No	+17.00DS/-2.00DC×95, +18.50DS/-1.00DC×60	Ophthalmic nystagmus	Esotropia
	II.1	0.7/1.0	Bilateral nuclear cataract	4 years	IOL	No	−0.50DS/-1.50DC×30，+1.50DS/-2.00DC×70	No	No
	III.1	1.0/1.0	Bilateral nuclear cataract	4 years	IOL	No	+1.75DS/-0.75DC×180, +2.00DS/-1.75DC×90	No	No

BCVA: best-corrected visual acuity. The best-corrected visual acuity was measured and calculated with the EDTRS visual acuity chart and represented with logMAR. Snellen equivalent is shown in the text. Bilateral nuclear cataract in family B has more opacity in the lens nucleus, while bilateral nuclear cataract in family A has less opacity. IOL: intraocular lens. NA: not acquired. NO: not found; R: right; L: left.

**Table 2:** Summary of probands sequencing data in three families.

Samples	Proband (family A)	Proband (family B)	Proband (family C)
Raw_data_bases (Mb)	2726.51	2825.35	1443.22
Clean_data_bases (Mb)	2615.89	2630.75	1393.44
Aligned_bases (Mb)	2597.73	2618.71	1387.15
Aligned (%)	99.31	99.54	99.55
Initial bases on target	456294	456294	455256
Base covered on target	453517	453355	451520
Coverage of target region (%)	99.39	99.36	99.18
Effective bases on target	177513627	189678089	127259738
Fraction of effective bases on target (%)	6.83	7.24	9.17
Average sequencing depth on target	389.03	415.69	279.53
Fraction of target covered with at least 4X (%)	99.16	99.09	98.73
Fraction of target covered with at least 10X (%)	98.95	98.78	97.89
Fraction of target covered with at least 20X (%)	98.28	98.01	95.63
Duplication rate (%)	22.72	24.99	28.34

**Table 3:** Summary of function prediction of three likely pathogenic variants.

Gene symbol	*CRYBB2* (family A)	*CRYBB2* (family B)	*CRYGD* (family C)
ID	chr22-25623876	chr22-25623876	chr2-208986446 208986447
Ref_Transcript	NM_000496	NM_000496	NM_006891
Exon	exon4	exon4	exon3
Nucleotide_Changes	c.230G>T	c.230G>A	c.475delG
Amino_Acid_Changes	p.G77V	p.G77D	p.A159Pfs∗9
Gene_Type	het	het	het
Pathogenic_Analysis	Uncertain	Uncertain	Likely pathogenic
clinvar	—	—	—
MutRatio	0.39	0.45	0.55
Mutation_Type	SNV	SNV	deletion
dbsnp	—	—	—
PathSNP	#N/A	#N/A	#N/A
MutInNormal	#N/A	#N/A	#N/A
1000Genome	#N/A	#N/A	#N/A
MutInDatabase	#N/A	#N/A	#N/A
1000g2015aug_all	—	—	—
ESP6500si	—	—	—
Inhouse	—	—	—
gnomAD_exome_ALL	—	—	—
gnomAD_exome_EAS	—	—	—
SIFT	0	0	—
SIFT_Predict	Damaging	Damaging	—
PolyPhen_2	0.999	0.999	—
PolyPhen_2_Predict	Probably_damaging	Probably_damaging	—
MutationTaster	1	1	—
MutationTaster_Predict	Disease_causing	Disease_causing	—
GERP++	5.08	5.08	—
GERP++_Predict	Conserved	Conserved	—
SPIDEX	0.5789	1.3267	—
REVEL_score	0.957	0.968	—
MCAP_score	0.157318302	0.219654258	—
MCAP_pred	P	P	—
InterVar	Likely pathogenic	Likely pathogenic	Uncertain_significance
Highest-MAF	—	—	—
Mygeno_InterACMG	PM2; PP3	PM2; PP3	PVS; PM2
Pathogenic_Analysis (based on ACMG guidlines)	Uncertain	Uncertain	Likely pathogenic

“#N/A” indicates that it does not exist in the database, and “-” indicates the frequency in the database. SIFT predictive value, the smaller the value, the more likely it is to cause disease. PolyPhen prediction value, the larger the value, the more likely it is to cause disease. MutationTaster prediction result, the larger the value, the more likely it is to cause disease. GERP++ indicates the value of predicting conservativeness among various species, and >2 indicates relatively conservative.

## Data Availability

The data used to support the findings of this study are included within the article.

## Supplementary Materials

Supplementary Materials Table S1 presents the 153 genes contained in the panel. Table S2 presents the evaluation of candidate variants for each family in silico analysis. The sequencing chromatograms of the three family members are shown in supplementary result 1, and the sequencing biological analysis results of the three families are shown in supplementary result 2.
