# Supplementary material for: Novel Likely Pathogenic Variants Identified by Panel-Based Exome Sequencing in Congenital Cataract Patients
Source: J Ophthalmol. 2021 Nov 17;2021:3847409. doi: 10.1155/2021/3847409 (PMC8612798; doi:10.1155/2021/3847409)
Supplement: Supplementary Materials — Table S1 presents the 153 genes contained in the panel. Table S2 presents the evaluation of candidate variants for each family in silico analysis. The sequencing chromatograms of the three family members are shown in supplementary result 1, and the sequencing biological analysis results of the three families are shown in supplementary result 2. [file 3847409.f1.zip › Supplementary Materials/Supplementary result 2 (1).docx]

Family A

TES was performed on the proband (III.1) and her parents (II.1, II.3). A total of 2726.51 Mb of raw data of average sequencing depth on target and 2615.89 Mb of clean data were obtained after TES. The coverage of all targeted regions was 99.31%. The designed targeted exomes in the 20× read were 98.28%, those in the 10× read were 98.95%, and those in the 4× read were 99.16%. After filtering out high-frequency variants in the database, 77 low-frequency variants were obtained. By comparison with previously reported genes associated with congenital cataracts, 5 candidate variants (*MTHFR*:p.K273R, *CYP1B1*:p.L107, *GDF6*:p.P85P, *EPG5*:p.S1083L, and *CRYBB2*:p.G77V) were screened out, and *CRYBB2*:p.G77V was identified by Sanger sequencing and co-segregation analysis (Figure 1c). Sanger sequencing revealed that *CRYBB2*: p.G77V was found only in the patients but not in other members of the family and 100 healthy control members (Figure 1d). The p.G77V change slightly increases hydrophobicity (Figure 1e) and has a mild effect on the structure. Multiple sequence alignment (DNAMAN, version 6.0.40) of multiple species revealed that the glycine at the 77th amino acid of CRYBB2 was highly conserved (Figure 1f). This mutation results in a valine-for-glycine substitution at amino acid residue 77 (G77V). PolyPhen-2 predicted the potential impact of the G77V mutation on the protein's structure and function to likely be deleterious (score = 0.999) (Figure 1g). PyMOL was used to visualize the structures of the wild-type and mutant CRYBB2 proteins, and the mutation did alter a tyrosine corner (Figure 1h).

Family B

TES was performed on the proband (III.1) and the parents (II.1, II.2). A total of 2825.35 Mb of raw data of average sequencing depth on target regions and 2630.75 Mb of clean data were obtained after TES. The coverage of all targeted regions was 99.54%. The designed targeted exomes in the 20× reads were 98.01%, those in the 10× reads were 98.78%, and those in the 4× reads were 99.09%. After filtering out the high-frequency variants in the database, 67 low-frequency variants were obtained. By comparison with genes previously reported to be associated with congenital cataracts, 4 candidate variants (*POMGNT1*:p.D584N, *FOXE3*: p.P53L, *PAX6*:c.*21_*20delAA, and *CRYBB2*: c.230G>A p.G77D) were screened out, and 1 variant, *CRYBB2*: c.230G>A p.G77D, was identified by Sanger sequencing and co-segregation analysis (Figure 2c). Sanger sequencing revealed that this likely pathogenic variant was found only in patients but not in other family members (Figure 2d). These data demonstrated that a novel variant, *CRYBB2*:c.230G>A (p.G77D), was related to family B. This variant resulted in an aspartic acid-for-glycine substitution at amino acid residue 77 (G77D). The mutant’s hydrophobicity was lower than that of the wild type (Figure 2e). Multiple sequence alignment of CRYBB2 from different species revealed that codon 77, where the p.G77D mutation occurred, was highly conserved (as indicated by the black arrow) (Figure 2f). PolyPhen-2 predicted the potential impact of the G77D mutation on the protein's structure and function to be likely deleterious (score = 0.999) (Figure 2g). PyMOL was used to visualize the structures of the wild-type and mutant CRYBB2 proteins (Figure 2h).

Family C

A total of 1443.22 Mb of raw data of average sequencing depth on targeted regions and 1393.44 Mb of clean data were obtained after TES. The coverage of all targeted regions was 99.55%. The designed targeted exomes in the 20× reads were 95.63%, those in the 10× reads were 97.89%, and those in the 4× reads were 98.73%. After filtering out the high-frequency variants in the database, 24 low-frequency variants were obtained. By comparison with previously reported genes associated with congenital cataracts, 4 candidate variants (*BFSP2*:c.379C>G p.Q127E, *BEST1*:c.20G>A p.S7N, *MIP*:c.319G>A p.V107I, and *CRYGD*:c.475delG p.A159Pfs*9) were screened out, and *CRYGD*:c.475delG p.A159Pfs*9 was identified by Sanger sequencing and co-separation analysis (Figure 3c). Sanger sequencing revealed that *CRYGD*: c.475delG p.A159Pfs*9 was found only in patients but not in other family members and 100 healthy controls (Figure 3d). These data indicated that a novel *CRYGD* (NM_006891.4) c.475delG, p.A159Pfs*9 variant located in the third exon of this gene was associated with family C. Multiple sequence alignment of CRYGD from different species showed that this sequence was highly conserved (Figure 3e). The identified variant, CRYGD:c.475delG p.A159Pfs*9, was generated by a frameshift mutation located 9 codons downstream that resulted in premature termination; this alteration resulted in a protein that was reduced to half the length of the full-length protein (Figure 3f) The protein model was visualized with PyMOL (Figure 3g), where the red portion indicates the truncated portion of the protein after the frameshift mutations.
