# Supplementary material for: Novel Likely Pathogenic Variants Identified by Panel-Based Exome Sequencing in Congenital Cataract Patients
Source: J Ophthalmol. 2021 Nov 17;2021:3847409. doi: 10.1155/2021/3847409 (PMC8612798; doi:10.1155/2021/3847409)
Supplement: Supplementary Materials — Table S1 presents the 153 genes contained in the panel. Table S2 presents the evaluation of candidate variants for each family in silico analysis. The sequencing chromatograms of the three family members are shown in supplementary result 1, and the sequencing biological analysis results of the three families are shown in supplementary result 2. [file 3847409.f1.zip › Supplementary Materials/Table S2.pdf]

	Gene_Symbol	ID	Ref_Transcript	Exon	Nucleotide_Chang	Amino_Acid_(	Gene_Type	1000g2015aug_all	Pathogenic_Analys	clinvar	MutRatio	Mutation_Type	dbsnp	PathSNP	MutInNorma	1000Genome	MutInDatal	1000g2015aug_all	ESP6500si	Inhouse	ExAC_ALL	ExAC_EAS	SIFT	SIFT_Predict	PolyPhen_2	PolyPhen_2_Predict	MutationTaster	MutationTaster_Predict	GERP++	GERP++_Predict	SPIDEX	REVEL_score	MCAP_score	MCAP_pred	InterVar	Highest-MAF	Mygeno_InterACMG	Pathogenic_Analysis
Family A	MTHFR	chr1-11855368	NM_005957	exon6	c.818A>G	p.K273R	het	–	Uncertain	–	0.52	SNV	–	#N/A	#N/A	#N/A	#N/A	–	–	–	–	–	0.059	Tolerated	0.038	Benign	1	Disease_causing	3.53	Conserved	-0.0865	0.368	0.071252519	P	Uncertain_	–	PM2	Uncertain
	CYP1B1	chr2-38302213	NM_000104	exon2	c.319C>G	p.L107V	het	0.000399361	Uncertain	–	0.55	SNV	rs56339482	#N/A	#N/A	chr2-38302213	#N/A	0.000399361	–	0.0021739	0.0006	0.0097	–	–	1	Probably_damaging	0.997	Disease_causing	2.73	Conserved	0.0907	0.647	0.783094831	P	Uncertain_	0.0097	PS1	Uncertain
	GDF6	chr8-97172666	NM_001001557	exon1	c.255G>T	p.P85P	het	0.00359425	Uncertain	–	0.52	SNV	rs112296824	#N/A	#N/A	chr8-97172666	#N/A	0.00359425	0.0041	0.0057	0.0015	0.0051	–	–	–	–	–	–	–	–	–	–	–	–	Benign	0.0057	BP7	Uncertain
	EPG5	chr18-43496539	NM_020964	exon18	c.3248C>T	p.S1083L	het	0.014976	Uncertain	–	0.45	SNV	rs78339727	#N/A	#N/A	chr18-43496539	#N/A	0.014976	0.0208	0.0286	0.029	0.0021	0.019	Damaging	0.771	Possibly_damaging	0.999	Disease_causing	5.78	Conserved	-0.2802	0.184	–	–	Benign	0.029	BS1	Uncertain
	CRYBB2	chr22-25623876	NM_000496	exon4	c.230G>T	p.G77V	het	–	Uncertain	–	0.39	SNV	–	#N/A	#N/A	#N/A	#N/A	–	–	–	–	–	0	Damaging	0.999	Probably_damaging	1	Disease_causing	5.08	Conserved	0.5789	0.957	0.157318302	P	Uncertain_	–	PM2;PP3	Likely pathogenic
Family B	POMGNT1	chr1-46655561	NM_001243766	exon20	c.1750G>A	p.D584N	het	–	Uncertain	–	0.53	SNV	–	#N/A	#N/A	#N/A	#N/A	–	–	–	–	–	0.389	Tolerated	0.079	Benign	1	Disease_causing	5.92	Conserved	-0.2323	0.077	0.015190102	B	Uncertain_	–	PM2;BP1	Uncertain
	FOXE3	chr1-47882145	NM_012186	exon1	c.158C>T	p.P53L	het	0.00658946	Uncertain	–	0.6	SNV	rs534479543	#N/A	#N/A	#N/A	#N/A	0.00658946	–	0.0114	–	–	0.159	Tolerated	0.013	Benign	1	Polymorphism	2.25	Conserved	–	0.113	–	–	Benign	0.0114	0	Uncertain
	PAX6	chr11-31811460 31811462	NM_001258464	–	c.*21_*20delAA	–	het	–	Uncertain	–	0.5	unknown	rs759391101	#N/A	#N/A	#N/A	#N/A	–	–	–	0.0009	0.0008	–	–	–	–	–	–	–	–	–	–	–	–	Uncertain_	0.0009	PM2	Uncertain
	CRYBB2	chr22-25623876	NM_000496	exon4	c.230G>A	p.G77D	het	–	Uncertain	–	0.45	SNV	–	#N/A	#N/A	#N/A	#N/A	–	–	–	–	–	0	Damaging	0.999	Probably_damaging	1	Disease_causing	5.08	Conserved	1.3267	0.968	0.219654258	P	Uncertain_	–	PM2;PP3	Likely pathogenic
Family C	BFSP2	chr3-133119306	NM_003571	exon1	c.379C>G	p.Q127E	het	0.00139776	Uncertain	–	0.41	SNV	rs185816798	#N/A	#N/A	chr3-133119306	#N/A	0.00139776	–	0.0108696	0.0008	0.0115	0.235	Tolerated	0.021	Benign	0.989	Disease_causing	3.81	Conserved	–	0.227	–	–	Benign	0.0115	BS1	Uncertain
	BEST1	chr11-61719298	NM_004183	exon2	c.20G>A	p.S7N	het	0.000199681	Likely pathogenic	–	0.57	SNV	rs199508634	#N/A	#N/A	chr11-61719298	#N/A	0.000199681	–	–	1.65E-05	0.0002	0.561	Tolerated	0.006	Benign	1	Disease_causing	-1.72	Nonconserved	0.1244	0.346	0.013177856	B	Uncertain_	0.0002	PS1;PM2;PM5;PM5	Likely pathogenic
	MIP	chr12-56848079	NM_012064	exon1	c.319G>A	p.V107I	het	0.0219649	Uncertain	–	0.5	SNV	rs74641138	#N/A	#N/A	chr12-56848079	#N/A	0.0219649	0.019	0.0065217	0.0253	0.0395	0.543	Tolerated	0.012	Benign	1	Disease_causing	4.29	Conserved	–	0.233	–	–	Benign	0.0395	PM1;BS1	Uncertain
	CRYGD	chr2-208986446 208986447	NM_006891	exon3	c.475delG	p.A159Pfs*9	het	–	Likely pathogenic	–	0.55	deletion	–	#N/A	#N/A	#N/A	#N/A	–	–	–	–	–	–	–	–	–	–	–	–	–	–	–	–	–	Uncertain_	–	PVS;PM2	Likely pathogenic
