# Supplementary figures and images for: Novel Likely Pathogenic Variants Identified by Panel-Based Exome Sequencing in Congenital Cataract Patients
Source: J Ophthalmol. 2021 Nov 17;2021:3847409. doi: 10.1155/2021/3847409 (PMC8612798; doi:10.1155/2021/3847409)

III-1

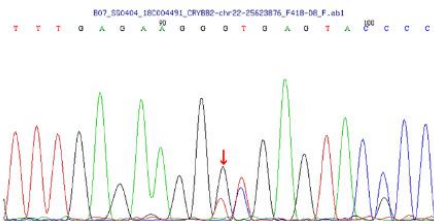

III-2

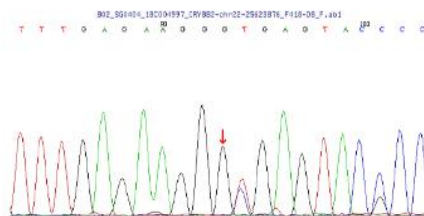

II-2

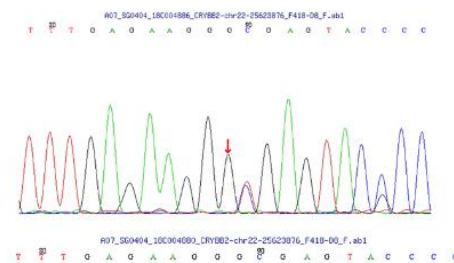

II-1

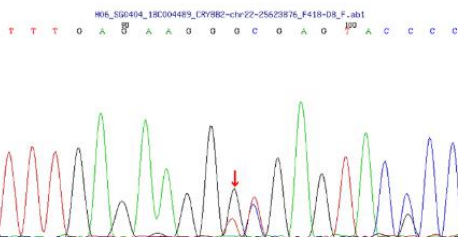

III-3

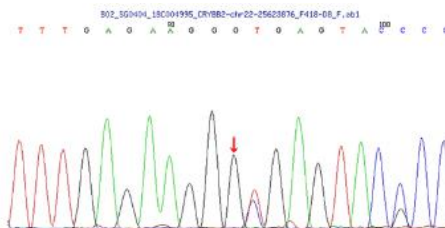

II-4

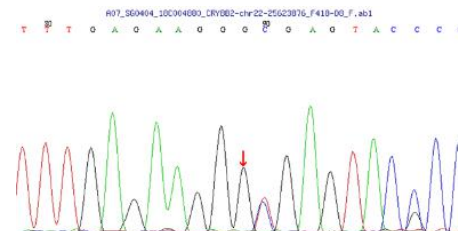

I-1

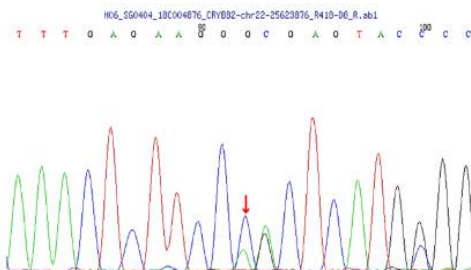

III-4

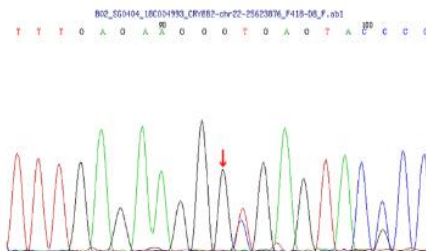

I-2

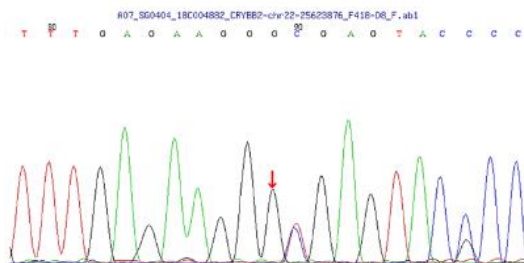

II-3

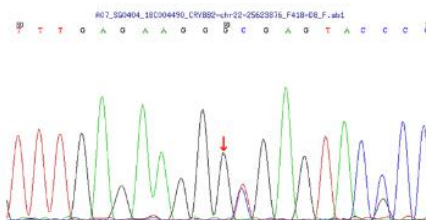

Supplement: Supplementary Materials — Table S1 presents the 153 genes contained in the panel. Table S2 presents the evaluation of candidate variants for each family in silico analysis. The sequencing chromatograms of the three family members are shown in supplementary result 1, and the sequencing biological analysis results of the three families are shown in supplementary result 2. [file 3847409.f1.zip › Supplementary Materials/Supplementary result 1/Sanger A_20211007164313.pdf]

III-1

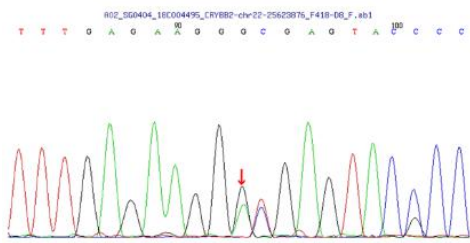

II-1

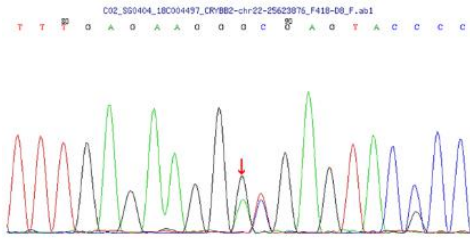

I-2

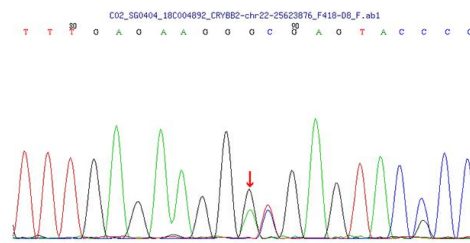

III-2

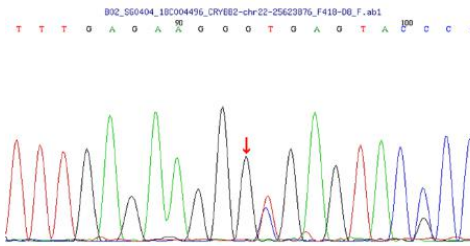

II-2

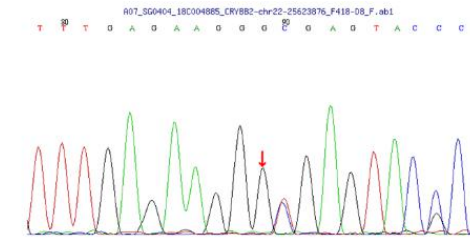

II-3

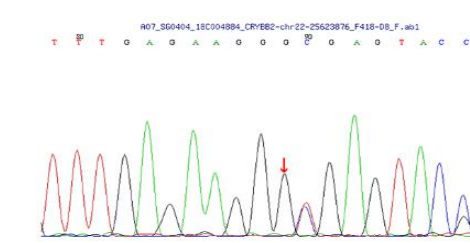

I-1

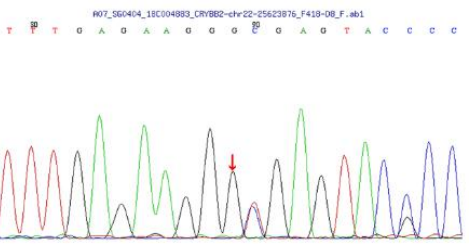

Supplement: Supplementary Materials — Table S1 presents the 153 genes contained in the panel. Table S2 presents the evaluation of candidate variants for each family in silico analysis. The sequencing chromatograms of the three family members are shown in supplementary result 1, and the sequencing biological analysis results of the three families are shown in supplementary result 2. [file 3847409.f1.zip › Supplementary Materials/Supplementary result 1/Sanger B_20211007164445.pdf]
